# When mentorship goes wrong: from personal vignettes to a system-level playbook for safer, fairer academic training

**DOI:** 10.3389/fmed.2025.1714113

**Published:** 2025-12-05

**Authors:** Bruno B. Andrade

**Affiliations:** 1 Division of Infectious Diseases, Department of Medicine, Johns Hopkins University, Baltimore, MD, United States; 2 Department of International Health, Bloomberg School of Public Health, Johns Hopkins University, Baltimore, MD, United States; 3 Laboratory of Clinical and Translational Research, Gonçalo Moniz Institute, Oswaldo Cruz Foundation, Salvador, Bahia, Brazil

**Keywords:** mentorship, sponsorship, Academic medicine, institutional reform, psychological safety, authorship, accountability

## 1 Introduction: Why this matters now

Mentorship is supposed to be ballast, a steady hand on the rudder while someone learns to sail in heavy weather. In medicine and science, we romanticize this role. We picture the senior clinician who opens doors, the principal investigator who fights for a stipend, the professor who notices a spark and fans it. We tell trainees to “find a mentor.” We rarely teach them what to do when the relationship curdles.

On paper, mentorship works. A systematic review in *JAMA* linked mentoring to career guidance, research productivity, and grant success, even while highlighting heterogeneity and methodological limits across studies (1). Qualitative work across academic health centers has mapped the features of both successful and failed pairings: clarity of expectations, aligned values, reciprocal commitment, and the mentor's willingness to spend social capital distinguish the former; opacity, misalignment, and one-sided commitment mark the latter (2).

Alongside these encouraging findings, a parallel literature documents negative mentoring experiences. Taxonomies of “harmful mentorship” describe neglect, manipulation, lack of expertise, and broader dysfunction (3). Team-level research on psychological safety shows how fear of embarrassment or retaliation silences questions and concerns, undermining learning precisely where stakes are high (4). In academic medicine, bullying, harassment, and discrimination are not rare outliers but documented, patterned phenomena that intersect with mentorship and training structures (5–7). Sponsorship, the active use of influence to advance a junior colleague, is also unevenly distributed, with women and underrepresented groups often receiving less advocacy even when mentored (8, 14).

Despite this accumulated knowledge, most institutional responses remain strangely generic: workshops, policy statements, aspirational values on websites. Trainees still lack a practical map for what to do when mentorship goes wrong: how to name what is happening, how to decide whether to repair, exit, or escalate, and what they can reasonably expect from institutions that claim to value mentoring.

I have been mentoring medical scientists, physicians, biotechnologists and biologists for over two decades, in several countries. In that time, I have celebrated students who flourished with generous guidance and honest feedback, but I have also watched talented people shrink, stall, or quietly leave because a mentoring relationship went wrong. Many of them did not complain formally; they came to my office, or caught me in a corridor, and told me stories that sounded uncomfortably similar, no matter the country, specialty, or institution. I have also had to confront my own blind spots as a mentor. Moments when, under pressure for grants, papers, and committees, I postponed meetings too often, answered emails too late, or failed to use my position to open doors for someone who trusted me. This piece grows out of that mix of privilege and discomfort: the privilege of seeing so many careers from close range, and the discomfort of recognizing how easily a system like ours can turn good intentions into harm. This Opinion article sits at that junction. It weaves together composite vignettes that many in academic medicine will recognize with an explicit decision pathway for trainees and a simple institutional framework, COMPASS, that links everyday mentoring practices to structural levers and concrete metrics. The goal is not to re-describe the problem, but to move from lament to a testable playbook for safer, fairer academic training.

## 2 The invisible cost of harmful mentorship

The damage caused by harmful mentorship is often quiet before it becomes dramatic. The literature captures this duality: on average, mentoring has modestly positive effects, but negative or neglectful relationships can reverse that benefit completely (1, 3, 5).

Consider a junior infectious diseases fellow paired with a renowned tuberculosis researcher. On paper, it looks ideal. The lab is high profile; the mentor is generous with ideas but largely absent in practice. Meetings slide, then disappear. Deadlines are flexible for the mentor but rigid for the fellow. Authorship remains a moving target discussed in passing, never in writing. Feedback arrives as late-night line edits with no real conversation about direction. No single episode feels catastrophic. Yet, by the end of the year, the fellow has a thin CV, a narrow skill set tied to the mentor's pipeline, and an expanding sense of dread. This is not cinematic abuse. It is neglect, and neglect is one of the most common negative mentoring experiences described by protégés (3).

The consequences extend beyond individual morale. When overwork, public shaming, or chronic unavailability substitute for guidance, team learning collapses. Psychological safety, the shared belief that it is safe to take interpersonal risks, is a prerequisite for the rapid error detection and adaptation that good science requires (4). In unsafe teams, people withhold dissent, soften inconvenient data, and keep nascent ideas to themselves. Science becomes more fragile just as the rhetoric about excellence becomes louder. Reviews of academic bullying in medical settings show how mistreatment correlates with burnout, mental health problems, and attrition, with trainees in oppressive environments more likely to abandon particular specialties or academic careers altogether (5).

The cost is also cumulative and material. It appears as stalled manuscripts, missed funding cycles, lost introductions, and delayed independence. Careers do not usually derail in one dramatic moment; they bend slowly under the weight of repeated, preventable failures of supervision, sponsorship, and safety.

## 3 A taxonomy of harm: What goes wrong and how it looks

Negative mentoring is not a single phenomenon. Classic work by Eby et al. organized protégés' reports into categories such as mentor distancing (being unavailable), manipulative behavior, lack of expertise, and general dysfunction (3). Subsequent studies have shown that these experiences are widespread; in some workplace samples, more than half of mentees report at least one negative mentoring event (3). Academic medicine adds its own layers of steep hierarchies, intense apprenticeship, and dependence on a few key gatekeepers (2, 5–7).

In practice, these patterns often appear as recognizable archetypes, summarized in Table 1, rather than as abstract categories:

- The Absentee Sponsor. Prestige is abundant; presence is scarce. Meetings are perpetually postponed, feedback is sporadic, and the mentee slowly internalizes the message that their development is peripheral. When deadlines slip, the cost is borne almost entirely by the trainee.
- The Extractor. Here, the mentee is treated less as a developing colleague and more as a convenient source of labor. Tasks multiply, the link to the mentee's goals becomes tenuous, and authorship remains vague until the moment of submission. Loyalty flows upward; credit does not.
- The Gatekeeper. Access becomes currency. Introductions, collaborations, and data are controlled in ways that primarily preserve the mentor's power. The mentee's visibility is rationed: conference invitations, committees, and awards are deferred indefinitely because “the timing is not right.”
- The Unsafe Teacher. Feedback wounds rather than sharpens. Errors are tallied; embarrassment is used as a teaching method. In such climates, curiosity shrinks and people stop taking intellectual risks. The day-to-day atmosphere violates the core features of psychological safety described in team research (4).
- The Boundary Crosser. Here, the problem is not misalignment but danger. Lines between professional and personal are blurred, and behavior crosses into harassment or discrimination. Surveys among surgical residents, for example, have documented high rates of gender discrimination and sexual harassment in training environments (7).

**Table 1:** Archetypes, failure modes, immediate actions, structural levers (COMPASS), and suggested metrics.

**Archetype**	**Failure mode (illustrative)**	**Immediate mentee action**	**Structural lever (COMPASS)**	**Suggested metrics**
Absentee Sponsor	Minimal contact; meetings repeatedly canceled; little feedback; no visible advocacy	Propose a compact with goals, timelines, and meeting cadence; request a co-mentor if engagement remains low	Clarity; Meetings; Opportunity; Accountability	Compact coverage; on-time meeting rates; number of sponsorship actions logged per mentee
Extractor	Heavy workload with tasks unrelated to mentee's development; ideas and data used without appropriate credit	Establish a prospective authorship and data-use plan; keep contribution logs; escalate if agreements are breached	Shared credit; Protection; Accountability	Proportion of mentee-first papers; visibility of contribution statements; time-to-resolution of disputes
Gatekeeper	Letters, introductions, collaborations, or data withheld; mentee's visibility consistently limited	Seek additional sponsors; involve program leadership in reviewing mentoring quality and access to opportunities	Opportunity; Accountability; Protection	Sponsorship logs; inclusion of mentoring and sponsorship quality in promotion decisions
Unsafe Teacher	Bullying, public shaming, microaggressions; climate undermines curiosity and learning	Document incidents; seek confidential advice; prioritize exit and, when appropriate, escalate through formal channels	Safety; Protection; Accountability	Climate and psychological safety indices; frequency and resolution timelines of reported mistreatment
Boundary Crosser	Inappropriate personal comments or contact; harassment or discrimination	Document specifics; use protected reporting mechanisms; request reassignment to a different supervisor or mentor	Protection; Safety; Accountability	Awareness of policies; ombudsperson access; case-closure metrics and evidence of non-retaliation

These archetypes do not cover every scenario, but they help trainees name what they are living. They also interact with structural harms that must be called out clearly. Sexual harassment and gender-based discrimination in academic medicine are tightly linked to concentrated power, weak accountability, and under-resourced reporting systems (6, 7). Sponsorship is itself structured by inequity: women and underrepresented groups are less likely to receive the active, public advocacy that moves careers forward, even when they are formally “well-mentored” (8, 14). A taxonomy that ignores these dynamics risks framing predictable design failures as individual fragility.

Recognizing the pattern is not about pathologizing imperfect mentors. It is about creating a shared language for behaviors that, left unnamed, silently shape who advances, who stalls, and who leaves.

## 4 Why systems produce bad mentoring: incentives, scarcity, and role conflation

If stories of “mentorship gone wrong” sound familiar across specialties and continents, it is because they are produced by structure as much as by personality. Academic medicine concentrates scarce rewards, grant funding, first and last authorship, protected time, prestige, inside supervisory relationships. At the same time, institutions often collapse three distinct roles into one person: the supervisor who controls data and funding, the evaluator who writes letters and signs off on progression, and the sponsor who opens doors.

This role conflation creates conflicts of interest even for well-intentioned people. Under conditions of grant scarcity and tournament-like competition for positions, rational actors may under-invest in mentoring, hoard credit, or limit a mentee's visibility to protect their own program (5–8, 14). The system, viewed from a distance, can resemble a pyramid: many trainees at the base, a narrowing set of secure posts at the top, and a culture in which everyone is encouraged to believe that exceptional effort alone will secure a place.

Three mechanisms recur in conversations with trainees and faculty. First, scarcity of time and funding converts mentoring into an optional luxury. Meetings are the first thing to go when deadlines loom, even though data show that structured mentoring contributes to productivity (1, 2, 9). Second, opaque norms around authorship, data access, and contribution statements make it easy for credit to flow toward those already in power. Third, the absence of explicit, written expectations about goals, timelines, and sponsorship leaves both parties improvising, which usually favors the person with more experience and leverage.

The result is a system in which negative mentoring is not an aberration but a predictable byproduct. Fixing this requires more than exhortations to “be a better mentor.” It requires dyadic tools that help pairs function better and institutional safeguards that separate incompatible roles, protect reporting, reward good mentoring, and make invisible work visible.

## 5 Recognizing a failing mentorship

Trainees often sense trouble long before they have language for it. The early signs are usually mundane. Meetings are repeatedly rescheduled and then quietly abandoned. Feedback, when it arrives, is non-specific (“keep working”) or purely editorial, with no discussion of direction or feasibility. Authorship plans remain verbal, resurface only at submission, and seem to shift with each iteration. Opportunities, talks, committees, collaborative calls, consistently flow toward the mentor, with little thought given to the mentee's visibility.

Qualitative studies in academic health centers describe how mentees in such relationships gradually reinterpret structural problems as personal failure (2). They start to believe they are “slow,” “not ready,” or “lucky to be here at all,” even when objective outputs suggest otherwise. Research on psychological safety helps explain this pattern: when people perceive interpersonal risk as high and their status as fragile, they self-silence (4). Questions remain unasked; concerns about fairness are downplayed; alternative mentors are not approached because it feels disloyal or dangerous.

A practical rule of thumb is to focus on trends rather than episodes. Every mentor will sometimes cancel a meeting or return a rough draft. What matters is the pattern over months: does the relationship move work forward in a measurable way? One simple practice is to keep contemporaneous notes of agreed tasks, deadlines, and responses. Put on one page what you were asked to do, what you delivered, and what came back. Patterns become easier to see when they are written down rather than felt in isolation.

Another signal is the body's own barometer. When the prospect of sending an email reliably produces dread, when you rehearse simple requests for days, when you carefully edit out any sentence that might sound like advocacy for your own development, the relationship is no longer a neutral container for learning. It has become a psychological stressor, and the literature on academic bullying suggests that prolonged exposure to such climates carries real risk (5).

## 6 A decision pathway for trainees: repair, exit, or escalate

Once a trainee recognizes that something is wrong, the next question is brutally simple: what now? Over years of discussing these situations with mentees and colleagues, I have found it useful to frame the options in three verbs: repair, exit, or escalate. These are not rigid steps but distinct moves, each with its own risks and evidence base.

### 6.1 Repair

Some misaligned relationships can be repaired, particularly when both parties are acting in good faith but swimming in institutional chaos. The most powerful tool here is specificity. Rather than a vague conversation about “needing more mentoring,” it is often more productive to bring a written, time-bound proposal: clear goals, concrete deliverables, timelines, and an initial authorship and data-use plan.

Randomized trials of mentor training in clinical and translational research demonstrate that focused curricula can improve communication, expectation-setting, and support for career development (9, 11). Instruments such as the Mentoring Competency Assessment (MCA) translate those skills into concrete domains, aligning expectations, maintaining effective communication, fostering independence, that can be discussed explicitly (10, 12). Mentees can adapt the same domains to frame their requests: regular meetings with agendas; agreed milestones; explicit discussion of authorship and sponsorship.

When a mentor engages sincerely with this process, adjusts meeting cadence, clarifies credit, follows through on agreed actions, the relationship may not only stabilize but strengthen. Not every poorly structured dyad is doomed. Some simply need the kind of deliberate scaffolding that we routinely build into clinical trials and rarely build into mentoring.

### 6.2 Exit

If repeated attempts at repair produce only temporary or cosmetic change, the problem may not be skills but incentives and bandwidth. In those cases, exit becomes an act of risk management rather than betrayal. Changing mentors is painful, particularly when trainees worry about being perceived as disloyal or ungrateful. Yet the cumulative opportunity cost of staying in a stagnant or exploitative relationship is often greater than the disruption of leaving.

One intermediate step is to diversify rather than immediately abandon: adding a co-mentor with complementary expertise, seeking an external sponsor, or joining a collaborative network that is not fully controlled by the current mentor. Multi-mentor models are increasingly encouraged for exactly this reason. Over time, as alternative sources of guidance and sponsorship solidify, the mentee can renegotiate or step away from the original dyad.

Empirical work reminds us that mentoring is, at best, one factor among many (1, 2). There is no virtue in persisting indefinitely in a relationship that consistently narrows rather than enlarges a trainee's trajectory.

### 6.3 Escalate

Escalation is different. When boundaries are crossed, harassment, discrimination, retaliation, plagiarism, the goal is not repair. It is protection and accountability. Reviews and commentaries on sexual harassment in academic medicine uncover how persistent these abuses are and how often victims face career risks when they speak up (5–7).

In these situations, documentation matters. Specific, dated accounts of who said or did what, in which setting, and with what impact, help move reports from vague impressions to actionable information. Trainees should not have to navigate this alone; confidential discussions with trusted faculty outside the immediate hierarchy, ombudspersons, or institutional offices can clarify options and protections. Institutions, for their part, must offer reporting channels that are independent, accessible, and backed by non-retaliation policies that are actually enforced (5–7).

Escalation is emotionally costly. But when safety is at stake, personal, psychological, or scientific, remaining silent shifts the entire burden of the system's failures onto the shoulders of those with the least power to fix them.

## 7 Institutional levers that work

Individual tactics, however thoughtful, cannot substitute for institutional responsibility. If academic systems reliably generate both extraordinary science and chronic mentoring failures, then the rules of the game need rewriting. The COMPASS framework in Box 1 offers one such rewrite. Its seven domains—Clarity, Opportunity, Meetings and micro-feedback, Protection and policies, Accountability, Shared credit, and Safety—pair everyday mentor–trainee practices with structural safeguards and simple metrics.

Box 1 **The COMPASS framework (checklist and indicative metrics)**

**COMPASS specifies seven domains that pair mentor–trainee practices with institutional safeguards and indicators**.

**Clarity**

For each active project, mentor and mentee agree on a brief written compact covering goals, primary outputs, milestones, meeting cadence, and a prospective authorship and data-use plan. A feasible indicator is the proportion of projects with an up-to-date compact and the share that include a written authorship plan.

**Opportunity (Sponsorship)**

Mentors commit to regular, logged advocacy actions such as introductions, invited talks, committee or award nominations, and visible credit in public forums. A practical indicator is a target number of documented sponsorship actions per mentee per year (e.g., three to four substantive actions), stratified by gender and other equity-relevant characteristics (8, 14).

**Meetings and micro-feedback**

Dyads schedule one-to-one meetings at a regular cadence, with simple agendas, decisions, and action items recorded in a shared document. Programs can monitor on-time meeting rates, frequency of documented check-ins, and completion of agreed action items as basic indicators of mentoring hygiene.

**Protection and policies**

Institutions provide confidential reporting channels; separate or counter-balance supervision, evaluation, and sponsorship roles where feasible; and ensure access to an independent ombudsperson. Awareness of these policies among trainees, the availability of ombudsperson support, and time-to-resolution for reported cases are relevant indicators of whether protections function in practice (5–7).

**Accountability**

Mentoring quality is incorporated into promotion and compensation via 360° mentoring evaluations and explicit recognition of protected mentoring time as part of full-time equivalent. Indicators include the use of mentoring scores in annual reviews, formal recognition of mentor training, and alignment between allocated and recorded mentoring time (9–13).

**Shared credit**

Prospective authorship criteria, transparent contribution statements (e.g., CRediT taxonomy), and clear data-use agreements are adopted at the departmental or program level. The proportion of mentee-first publications, visibility of contribution statements in journals and institutional reports, and satisfaction with credit allocation in climate surveys serve as indicators.

**Safety (psychological)**

Programs conduct periodic climate assessments that explicitly ask about psychological safety, exposure to bullying or harassment, confidence in reporting mechanisms, and perceived fairness of authorship and sponsorship. Trends in a safety index, coupled with documented follow-through on identified problems, provide practical signals of whether the environment is becoming safer over time (4–8, 11–13).

Clarity begins with written mentorship compacts for each active project. These are not legal documents but shared maps: goals, milestones, meeting cadence, and a prospective authorship and data-use plan. A straightforward indicator is coverage: what proportion of active projects have a compact, and how often are these revisited?

Opportunity (Sponsorship) recognizes that advice without advocacy is incomplete. Institutions can normalize and track sponsorship actions, introductions, nominations, invitations to speak or co-chair, expecting mentors to document, for each mentee, a minimum number per year. Surveys and scoping reviews of sponsorship in academic medicine support the link between active advocacy and career progression, as well as the current inequities in who receives such advocacy (8, 14).

Meetings and micro-feedback sound unexciting but often determine whether work moves or stalls. Programs can encourage scheduled one-to-one meetings with simple agendas and shared notes, and they can monitor on-time meeting rates and closure of action items as basic indicators of mentoring hygiene.

Protection and policies require more structural investment: confidential reporting channels; separation or counter-balancing of supervision, evaluation, and sponsorship roles; access to an independent ombudsperson. Time-to-resolution metrics for reported cases and awareness of policies among trainees are tangible signals of whether these protections exist in practice rather than only on paper (5–7).

Accountability means that mentoring quality is not treated as a private craft but as part of formal evaluation. 360° mentoring evaluations, aggregated to protect individuals, can inform promotion and compensation decisions. Several randomized and cohort studies have shown that mentor training can improve competencies (9, 11–13); pairing such training with evaluations and explicit protected time for mentoring (as part of FTE allocation) closes the loop between expectation and reward.

Shared credit addresses authorship and visibility. Prospective authorship criteria, contribution statements, and transparent data-use agreements reduce disputes and curb the Extractor and Gatekeeper archetypes. Simple metrics, the share of mentee-first papers, visibility of contribution statements, can be tracked at the departmental level.

Safety (psychological) is sustained through regular climate assessments that explicitly ask about mentoring, sponsorship, and bystander confidence in reporting mechanisms. Psychological safety, originally examined in industrial and clinical teams (4), can be monitored in academic units as well, with documented follow-through when problems are identified.

Taken together, these levers form an evaluable bundle rather than an aspirational wish list. Departments and programs can adopt a subset within an academic year, collect baseline metrics, and test whether negative-mentoring reports fall, mentee visibility increases, and perceived safety improves. Box 1 outlines COMPASS in more operational detail whereas Table 1 links the earlier archetypes of harmful mentorship to specific trainee actions, structural levers, and suggested metrics.

## 8 Limitations and scope

This is an Opinion article grounded in published evidence and lived experience, not a systematic review. The vignettes are composites, drawn from multiple conversations and contexts, to protect identities while illustrating recurring patterns. The decision pathway, repair, exit, escalate, is intentionally simple, designed as a starting point for trainees rather than a comprehensive algorithm. Similarly, COMPASS focuses on feasible, auditable steps that departments and programs could realistically implement and study within existing constraints.

These proposals should be formally evaluated. Future work might test the impact of mentorship compacts, sponsorship logs, and 360° evaluations on objective outcomes such as time to promotion, publication patterns, and attrition, as well as on psychological safety and perceived fairness. The fact that mentoring failures are structurally patterned means that system-level experiments are both possible and necessary.

## 9 Conclusion

When mentorship goes wrong, it is tempting to tell the story as a clash of personalities: the brilliant but distracted supervisor, the demanding mentee, the unfortunate mismatch. The evidence paints a different picture. Harmful mentoring is often the visible symptom of deeper design choices, who holds power over whom, how credit is allocated, what behaviors are rewarded or ignored.

The decision pathway outlined here gives trainees a way to move from confusion to action: first, to attempt repair with specific, written expectations; then, if needed, to exit relationships that consistently erode their development; and, when safety is compromised, to escalate through protected channels. COMPASS extends that logic upstream, specifying how institutions can make good mentoring easier and bad mentoring harder: compacts that create clarity, logged sponsorship that spreads opportunity, routines that normalize feedback, protections that make reporting less perilous, accountability that values mentoring as real work, authorship policies that share credit, and a climate that takes psychological safety as seriously as scientific rigor.

Many of us who now supervise others carry our own memories of mentors who showed up, advocated even when it cost them something, shared authorship generously, and corrected us in ways that preserved dignity. We also remember, often just as vividly, the experiences that made us smaller. The task now is to move beyond private vows to “do better” and to build systems in which mentoring that is fair, honest, and generative is not an act of individual heroism but the expected norm.
